# Diaqua­{2,6-bis­[*N*-(2-pyridinylmeth­yl)­carbamo­yl]­phenolato-κ^2^ *O* ^1^,*O* ^2^}zinc(II)

**DOI:** 10.1107/S1600536808016693

**Published:** 2008-06-07

**Authors:** Chomchai Suksai, Sarayut Watchasit, Thawatchai Tuntulani, Chaveng Pakawatchai

**Affiliations:** a Department of Chemistry, Faculty of Science, Burapha University, Chonburi 20131, Thailand; b Department of Chemistry, Faculty of Science, Chulalongkorn University, Bangkok 10330, Thailand; c Department of Chemistry, Faculty of Science, Prince of Songkla University, Songkhla 90112, Thailand

## Abstract

In the title compound, [Zn(C_20_H_17_N_4_O_3_)_2_(H_2_O)_2_], the Zn^II^ atom, lying on a twofold rotation axis, is six-coordinated in a distorted octa­hedral geometry by two phenolate O atoms and two carbonyl O atoms from two 2,6-bis­[(pyridin-2-ylmeth­yl)­carbamo­yl]phenolate ligands and by two water mol­ecules. A three-dimensional network is built up from an extensive array of hydrogen bonds and π–π inter­actions between the pyridyl rings, with a centroid–centroid distance of 3.666 (3) Å.

## Related literature

For related literature, see: Chaudhuri *et al.* (2007 ▶); Goldsmith *et al.* (2002 ▶); Gumbley & Stewart (1984 ▶); Ingle *et al.* (2007 ▶); Kimura (1994 ▶); Lipscomb & Sträter (1996 ▶); Szajna-Fuller *et al.* (2007 ▶).
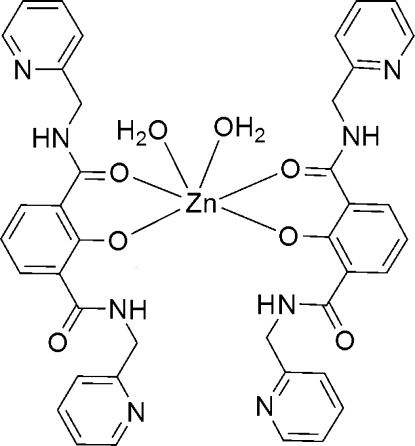


## Experimental

### 

#### Crystal data

[Zn(C_20_H_17_N_4_O_3_)_2_(H_2_O)_2_]
*M* *_r_* = 824.18
Monoclinic, 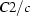

*a* = 16.357 (4) Å
*b* = 14.723 (4) Å
*c* = 15.135 (4) Å
β = 91.938 (7)°
*V* = 3642.9 (16) Å^3^
*Z* = 4
Mo *K*α radiation
μ = 0.74 mm^−1^
*T* = 293 (2) K
0.35 × 0.3 × 0.2 mm

#### Data collection

Bruker SMART APEX CCD area-detector diffractometer
Absorption correction: multi-scan (*SADABS*; Sheldrick, 1996 ▶) *T*_min_ = 0.757, *T*_max_ = 0.854
21455 measured reflections
4398 independent reflections
3575 reflections with *I* > 2σ(*I*)
*R*_int_ = 0.061

#### Refinement

*R*[*F* ^2^ > 2σ(*F* ^2^)] = 0.060
*wR*(*F* ^2^) = 0.130
*S* = 1.11
4398 reflections
260 parameters
H-atom parameters constrained
Δρ_max_ = 0.52 e Å^−3^
Δρ_min_ = −0.42 e Å^−3^

### 

Data collection: *SMART* (Bruker, 2007 ▶); cell refinement: *SAINT* (Bruker, 2007 ▶); data reduction: *SAINT*; program(s) used to solve structure: *SHELXS97* (Sheldrick, 2008 ▶); program(s) used to refine structure: *SHELXL97* (Sheldrick, 2008 ▶); molecular graphics: *ORTEP-3* (Farrugia, 1997 ▶) and *Mercury* (Macrae *et al.*, 2006 ▶); software used to prepare material for publication: *SHELXL97*.

## Supplementary Material

Crystal structure: contains datablocks global, I. DOI: 10.1107/S1600536808016693/hy2136sup1.cif

Structure factors: contains datablocks I. DOI: 10.1107/S1600536808016693/hy2136Isup2.hkl

Additional supplementary materials:  crystallographic information; 3D view; checkCIF report

## Figures and Tables

**Table:** 

O1—Zn1	1.9772 (18)
O2—Zn1	2.1572 (19)
O4—Zn1	2.149 (2)

**Table:** 

O1—Zn1—O1^i^	175.44 (11)
O1—Zn1—O4^i^	85.97 (8)
O1—Zn1—O4	97.42 (8)
O4^i^—Zn1—O4	84.55 (12)
O1—Zn1—O2	84.29 (7)
O4—Zn1—O2	91.26 (8)
O1—Zn1—O2^i^	92.61 (8)
O4—Zn1—O2^i^	168.82 (8)
O2—Zn1—O2^i^	94.67 (12)

Symmetry code: (i) 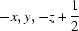.

**Table 2:** Hydrogen-bond geometry (Å, °)

*D*—H⋯*A*	*D*—H	H⋯*A*	*D*⋯*A*	*D*—H⋯*A*
N3—H3*A*⋯O1	0.86	1.93	2.623 (3)	136
N1—H1⋯N4^ii^	0.86	2.20	3.007 (4)	155
O4—H24⋯O3^iii^	0.85	1.87	2.712 (3)	174
O4—H23⋯N2^iv^	0.87	2.03	2.879 (3)	163

Symmetry codes: (ii) 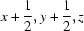; (iii) 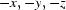; (iv) 
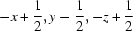
.

**Fig. 1.**
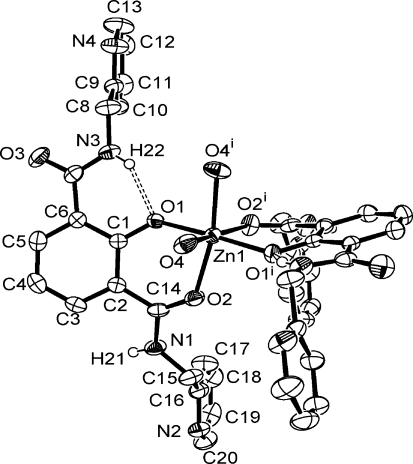
The molecular structure of the title compound. Displacement ellipsoids are drawn at the 50% probability level. H atoms have been omitted except those involved in hydrogen bonds. Hydrogen bonds are shown as dashed lines. [Symmetry code: (i) -x, y, 0.5 - z.]

**Fig. 2.**
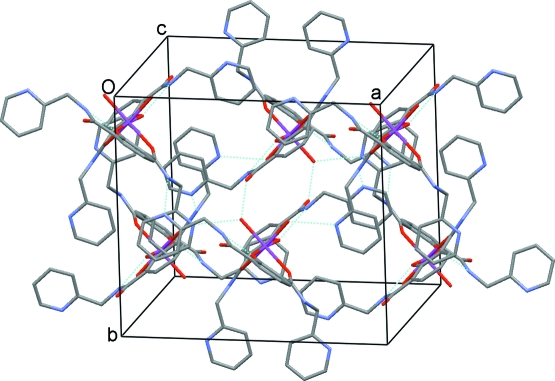
The three-dimensional hydrogen bonding network in the title compound. H atoms have been omitted for clarity. Hydrogen bonds are shown as dashed lines.

**Fig. 3.**
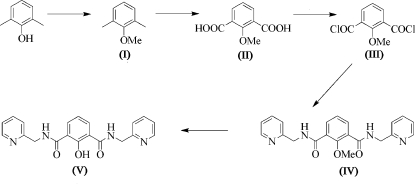
The synthesis of compounds (IV) and (V).

## supplementary crystallographic information

### Comment

Zinc complexes of common amide-containing ligands have been widely explored and have received much attention in biomimetic research (Ingle *et al.*, 2007). For example, the zinc complex with 6-(pivaloylamido-2-pyridylmethyl)amine ligand has been synthesized to serve as models for amide hydrolysis activity (Szajna-Fuller *et al.*, 2007). Recently, zinc and copper complexes with a family of pyridylmethylamide ligands have been synthesized and showed that these ligands coordinate to metal ions with different coordination modes (Chaudhuri *et al.*, 2007). It should be mentioned that zinc complexes containing aqua ligands have been used as model studies for zinc-hydrolase enzyme because of the functional unit at the active site of zinc-hydrolase enzyme is a zinc-bound water molecule, which is deprotonated at near neutral pH to generate a strongly nucleophilic Zn—OH group (Lipscomb & Sträter, 1996). Moreover, the aqua ligand bound to zinc ion is further stabilized *via* the formation of a hydrogen bond with a carboxylate or phenolic group from amino acid residues (Kimura, 1994). This hydrogen bond has been postulated to play a role in the activation of the coordinated aqua ligand in the catalytic pathway.

In an ongoing effort to study the interaction of zinc ion with aqua ligand, we report here the synthesis and characterization of the title compound, a new zinc complex with 2,6-bis[(pyridin-2-ylmethyl)carbamoyl]phenolate and aqua ligands. The electrospray mass spectrometry (ESI-MS) of the title compound confirmed the presence of the molecular species in solution. The compound has fragmentation patterns with peaks at m/*z* = 823.17 and 787.19; the former corresponds to the [*M*+2H_2_O+H^+^] ion and the latter is consistent with the loss of two coordinated water molecules [*M*–2H_2_O+H^+^]. The evidence for the presence of water in the complex is also given by IR absorption at 3568 cm^-1^. The elemental analysis agrees well with the proposed structure.

In the title compound, the Zn^II^ atom is situated on a twofold rotation axis in a distorted octahedral coordination geometry, which is defined by two phenolate O atoms, two carbonyl O atoms and two *cis* water molecules (Fig. 1). The O1—Zn1—O1^i^ and O4—Zn1—O2^i^ [symmetry code: (i) -*x*, *y*, 1/2 - *z*] angles deviate from linearity (Table 1). These results are in accordance with the distorted octahedral geometry. It is noteworthy that each ligand behaves in a bidentate coordination fashion involving one phenolate O atom and one carbonyl O atom, while the two amide N atoms and pyridyl N atoms are free of coordination with the Zn atom. The two phenyl rings of coordinated molecules are tilted to one another with a dihedral angle of 72.3 (3)°. Remarkably, the intramolecular N3—H3A···O1 hydrogen bond forms a pseudo-six-membered ring (Fig. 1).

The water molecule is involved in an extensive intermolecular hydrogen-bonding network, as shown in Fig. 2. Atom O4 of aqua ligand acts as a hydrogen-bond donor to the uncoordinated carbonyl O3 atom and uncoordinated pyridyl N2 atom of adjacent complex molecules, respectively (Table 2). Additionally, the molecules are held together by intermolecular hydrogen bond between the uncoordinated amide N1 atom and uncoordinated pyridyl N4 atom. Not only the intermolecular hydrogen bonds, but also there are intermolecular π–π interactions in the crystal structure, which occur between the pyridyl rings containing atoms N2 and N4 of adjacent molecules, with a centroid–centroid distance of 3.666 (3) Å.

### Experimental

Intermediate products (I), (II) (Gumbley & Stewart, 1984) and (III) (Goldsmith *et al.*, 2002) have been synthesized in accordance with the published procedures. The methods to synthesize compounds (IV) and (V) are shown in Fig. 3.

A methanol solution (5 ml) of Zn(ClO_4_)_2_.6H_2_O (2.22 g, 5.96 mmol) was added dropwise to a stirred solution of compound (V) (1.00 g, 2.98 mmol) in methanol (10 ml) at room temperature and the solution was stirred for 3 h. Water (10 ml) was added to the solution to precipitate a white solid. The precipitate was filtered off and washed with water to obtain the white powder of the title compound (yield 28%, 0.66 g). m.p. 170–173 °C. Recrystallization of this powder in methanol yielded colourless block crystals of the title compound, suitable for X-ray diffraction study. Analysis, calculated for C_40_H_38_N_8_O_8_Zn: C 58.29, H 4.65, N 13.60%; found: C 58.20, H 4.67, N 13.61%. ^1^H-NMR (400 MHz, DMSO-*d*_6_): δ 10.95 (bs, 2H, –NH), 8.50 (d, *J* = 4.0 Hz, 2H, ArH), 7.95 (s, 2H, ArH), 7.74 (t, *J* = 7.6 Hz, 2H, ArH), 7.33 (t, *J* = 8.0 Hz, 2H, ArH), 7.26 (t, *J* = 5.6 Hz, 2H, ArH), 6.60 (bs,1*H*, ArH), 4.60 (s, 2H, –CH_2_–), 4.59 (s, 2H,–CH_2_–). ^13^C-NMR (100 MHz, DMSO-*d*_6_): δ 168.97, 159.20, 149.25, 137.39, 133.99, 122.61, 121.66, 111.55, 44.73. ESI-MS: m/*z* 787.19 [M–2H_2_O+H^+^], 823.17 [*M*+2H_2_O+H^+^].

### Refinement

H atoms on C and N atoms were positioned geometrically and refined as riding atoms, with C—H = 0.93 (aromatic CH), 0.97 (CH_2_) Å and N—H = 0.86 Å and *U*_iso_(H) = 1.2*U*_eq_(C,*N*). H atoms attached to the water molecule were found in difference Fourier map and refined isotropically with atomic coordinates fixed.

### Crystal data

**Table:**

[Zn(C_20_H_17_N_4_O_3_)_2_(H_2_O)_2_]	*F*_000_ = 1712
*M**_r_* = 824.18	*D*_x_ = 1.503 Mg m^−^^3^
Monoclinic, *C*2/*c*	Mo *K*α radiation λ = 0.71073 Å
Hall symbol: -C 2yc	Cell parameters from 4398 reflections
*a* = 16.357 (4) Å	θ = 1.9–28.0º
*b* = 14.723 (4) Å	µ = 0.74 mm^−^^1^
*c* = 15.135 (4) Å	*T* = 293 (2) K
β = 91.938 (7)º	Prism, colourless
*V* = 3642.9 (16) Å^3^	0.35 × 0.3 × 0.2 mm
*Z* = 4	

### Data collection

**Table:**

Bruker SMART APEX CCD area-detector diffractometer	3575 reflections with *I* > 2σ(*I*)
Monochromator: graphite	*R*_int_ = 0.061
*T* = 293(2) K	θ_max_ = 28.0º
φ and ω scans	θ_min_ = 1.9º
Absorption correction: multi-scan(SADABS; Sheldrick, 1996)	*h* = −21→21
*T*_min_ = 0.757, *T*_max_ = 0.854	*k* = −19→19
21455 measured reflections	*l* = −19→19
4398 independent reflections	

### Refinement

**Table:**

Refinement on *F*^2^	Secondary atom site location: difference Fourier map
Least-squares matrix: full	Hydrogen site location: inferred from neighbouring sites
*R*[*F*^2^ > 2σ(*F*^2^)] = 0.060	H-atom parameters constrained
*wR*(*F*^2^) = 0.130	*w* = 1/[σ^2^(*F*_o_^2^) + (0.0553*P*)^2^ + 3.7977*P*] where *P* = (*F*_o_^2^ + 2*F*_c_^2^)/3
*S* = 1.11	(Δ/σ)_max_ < 0.001
4398 reflections	Δρ_max_ = 0.52 e Å^−^^3^
260 parameters	Δρ_min_ = −0.42 e Å^−^^3^
Primary atom site location: structure-invariant direct methods	Extinction correction: none

### Special details

**Table:**

Experimental. Compound (IV): To a solution of (III) (2.38 g, 10.2 mmol) in dry CH_2_Cl_2_ (5 ml) was added to a well stirred mixture of 2-(aminomethyl)-pyridine (2.60 ml, 25.5 mmol) and NEt_3_ (5.33 ml, 38.3 mmol) in dried CH_2_Cl_2_ (5 ml) under nitrogen atmosphere and the reaction was then left stirring overnight. Next, the solvent was removed under vacuum and the residue was purified by column chromatography on Al_2_O_3_ with 50% EtOAc:CH_2_Cl_2_ as eluent. The resulting pale yellow solid was recrystallized in diethyl ether to give a pure white solid (IV) (yield 68%, 2.68 g). m.p. 120–125 °C. ^1^H-NMR (400 MHz, CDCl_3_): δ 8.70 (s, 2H, –NH), 8.61 (d, *J* = 4.4 Hz, 2H, ArH), 8.20 (d, *J* = 8.0 Hz, 2H, ArH), 7.71 (m, 2H, PyH), 7.38 (m, 3H, ArH), 7.25 (m, 2H, ArH), 4.85 (s, 2H, –CH_2_–), 4.83 (s, 2H, –CH_2_–), 3.88 (s, 3H, –CH_3_). ^13^C-NMR (100 MHz, DMSO-d_6_): δ 164.90, 156.69, 156.53, 149.18, 136.81, 134.82, 127.60, 125.11, 122.45, 122.24, 63.83, 45.16.Compound (V): Anhydrous LiI (3.89 g, 28.9 mmol) was added to a well stirred solution of (IV) (0.78 g, 2.89 mmol) in anhydrous pyridine (20 ml) at room temperature. The reaction was allowed to proceed for 7 d with constant stirring. Then pyridine was removed in *vacuum* and the residue was dissloved in 1 *M* HCl (20 ml) and extracted with ethyl acetate (3 *x* 20 ml). The combined organic phase was dried over anhydrous Na_2_SO_4_, filtered and brought to dryness by rotary evaporation. The crude product was recrystallized in a solution of methanol and diethyl ether, giving (V) as a white solid (yield 92% ,0.95 g). m.p 100–103 °C. Analysis, calculated for C_20_H_18_N_4_O_3_: C 66.29, H 5.01, N 15.46%; found: C 66.31, H 4.99, N 15.45%. ^1^H-NMR (400 MHz, CDCl_3_): δ 8.77(s, 2H, –NH), 8.61 (d, *J* = 4.8 Hz, 2H, Ar*H*), 8.11 (d, *J* = 7.6 Hz, 2H, ArH), 7.71 (m, 2H, ArH), 7.39 (d, *J* = 7.6 Hz, 2H, ArH), 7.26 (m, 2H, ArH), 7.03 (t, *J* = 8.0 Hz, 1H, ArH), 4.82 (s, 2H, –CH_2_–), 4.81 (s, 2H, –CH_2_–). ^13^C-NMR (100 MHz, CDCl_3_): δ 167.66, 160.59, 156.26, 149.06, 137.01, 133.28, 122.52, 122.08, 118.61, 117.94, 44.73. ESI: m/z 348.1458 [M+H^+^].

### Fractional atomic coordinates and isotropic or equivalent isotropic displacement parameters (Å^2^)

**Table:**

	*x*	*y*	*z*	*U*_iso_*/*U*_eq_	
C1	0.02286 (15)	0.19100 (17)	0.05522 (17)	0.0236 (5)	
C2	0.09384 (16)	0.24793 (18)	0.06360 (17)	0.0269 (6)	
C3	0.14191 (17)	0.26133 (19)	−0.0097 (2)	0.0343 (6)	
H3	0.1885	0.2972	−0.0036	0.041*	
C4	0.12260 (19)	0.2232 (2)	−0.0905 (2)	0.0403 (7)	
H4	0.156	0.2329	−0.1382	0.048*	
C5	0.05325 (18)	0.1705 (2)	−0.10030 (19)	0.0368 (7)	
H5	0.0395	0.1457	−0.1553	0.044*	
C6	0.00339 (16)	0.15366 (17)	−0.02945 (17)	0.0264 (6)	
C7	−0.06868 (17)	0.09354 (18)	−0.04851 (19)	0.0312 (6)	
C8	−0.17697 (17)	0.0002 (2)	0.0089 (2)	0.0389 (7)	
H8A	−0.183	−0.0314	0.0645	0.047*	
H8B	−0.1619	−0.0445	−0.0347	0.047*	
C9	−0.25903 (17)	0.04027 (19)	−0.01925 (18)	0.0299 (6)	
C10	−0.27189 (18)	0.1306 (2)	−0.0387 (2)	0.0377 (7)	
H10	−0.2288	0.1718	−0.0348	0.045*	
C11	−0.3496 (2)	0.1594 (2)	−0.0642 (2)	0.0461 (8)	
H11	−0.3598	0.2203	−0.0764	0.055*	
C12	−0.4113 (2)	0.0968 (3)	−0.0711 (2)	0.0539 (9)	
H12	−0.4638	0.1139	−0.0897	0.065*	
C13	−0.3936 (2)	0.0081 (3)	−0.0498 (3)	0.0578 (10)	
H13	−0.4358	−0.0343	−0.0539	0.069*	
C14	0.11584 (15)	0.29456 (18)	0.14819 (19)	0.0291 (6)	
C15	0.1960 (2)	0.4212 (2)	0.2162 (2)	0.0428 (8)	
H15A	0.2548	0.4131	0.2219	0.051*	
H15B	0.1723	0.3991	0.2701	0.051*	
C16	0.17668 (18)	0.5211 (2)	0.20506 (19)	0.0352 (6)	
N2	0.23880 (15)	0.57968 (17)	0.21397 (17)	0.0372 (6)	
C20	0.2219 (2)	0.6684 (2)	0.2034 (2)	0.0478 (8)	
H20	0.2647	0.7097	0.2104	0.057*	
C19	0.1456 (3)	0.7013 (3)	0.1829 (2)	0.0544 (9)	
H19	0.137	0.7633	0.1753	0.065*	
C18	0.0825 (2)	0.6416 (3)	0.1738 (3)	0.0615 (10)	
H18	0.0298	0.6619	0.1599	0.074*	
N1	0.16417 (16)	0.36823 (17)	0.14154 (17)	0.0383 (6)	
H1	0.1771	0.3851	0.0894	0.046*	
C17	0.0979 (2)	0.5508 (3)	0.1857 (3)	0.0553 (9)	
H17	0.0553	0.5091	0.1806	0.066*	
N3	−0.11105 (14)	0.06472 (16)	0.01965 (16)	0.0339 (5)	
H3A	−0.0987	0.0852	0.0716	0.041*	
N4	−0.31909 (16)	−0.02078 (19)	−0.02365 (19)	0.0458 (7)	
O1	−0.02465 (11)	0.17550 (13)	0.12131 (12)	0.0297 (4)	
O2	0.09257 (12)	0.26946 (14)	0.22198 (13)	0.0363 (5)	
O3	−0.08908 (14)	0.07239 (16)	−0.12543 (15)	0.0473 (6)	
O4	0.08829 (13)	0.06216 (15)	0.24782 (15)	0.0431 (5)	
Zn1	0	0.17016 (3)	0.25	0.02664 (14)	
H24	0.0847	0.0211	0.2086	0.052 (11)*	
H23	0.1415	0.067	0.2476	0.078 (14)*	

### Atomic displacement parameters (Å^2^)

**Table:**

	*U*^11^	*U*^22^	*U*^33^	*U*^12^	*U*^13^	*U*^23^
C1	0.0218 (12)	0.0203 (12)	0.0284 (13)	0.0028 (10)	−0.0027 (10)	0.0033 (10)
C2	0.0270 (13)	0.0237 (13)	0.0299 (14)	0.0008 (11)	0.0002 (11)	0.0035 (11)
C3	0.0272 (14)	0.0312 (15)	0.0447 (17)	−0.0039 (12)	0.0047 (12)	0.0051 (13)
C4	0.0400 (17)	0.0441 (18)	0.0376 (17)	0.0014 (14)	0.0120 (13)	0.0059 (14)
C5	0.0395 (16)	0.0410 (17)	0.0299 (15)	0.0054 (14)	0.0015 (12)	−0.0021 (13)
C6	0.0261 (13)	0.0243 (14)	0.0286 (13)	0.0041 (10)	−0.0012 (10)	0.0015 (10)
C7	0.0315 (14)	0.0255 (14)	0.0362 (16)	0.0068 (11)	−0.0054 (12)	−0.0068 (12)
C8	0.0300 (15)	0.0307 (16)	0.055 (2)	−0.0043 (12)	−0.0110 (13)	0.0031 (14)
C9	0.0305 (14)	0.0292 (14)	0.0296 (14)	−0.0046 (11)	−0.0042 (11)	0.0031 (11)
C10	0.0344 (16)	0.0315 (15)	0.0472 (18)	−0.0007 (13)	−0.0008 (13)	0.0027 (13)
C11	0.0454 (19)	0.0428 (19)	0.050 (2)	0.0122 (15)	−0.0002 (15)	0.0050 (15)
C12	0.0333 (17)	0.068 (2)	0.060 (2)	0.0082 (17)	−0.0099 (15)	0.0120 (19)
C13	0.0321 (17)	0.062 (2)	0.078 (3)	−0.0144 (17)	−0.0142 (17)	0.019 (2)
C14	0.0208 (13)	0.0247 (13)	0.0412 (16)	0.0002 (10)	−0.0071 (11)	0.0041 (12)
C15	0.0442 (18)	0.0369 (17)	0.0462 (19)	−0.0125 (14)	−0.0135 (14)	0.0017 (14)
C16	0.0353 (16)	0.0353 (16)	0.0346 (16)	−0.0068 (13)	−0.0049 (12)	0.0002 (12)
N2	0.0360 (13)	0.0322 (13)	0.0427 (14)	−0.0078 (11)	−0.0071 (11)	−0.0002 (11)
C20	0.059 (2)	0.0314 (16)	0.052 (2)	−0.0095 (16)	−0.0073 (16)	−0.0007 (15)
C19	0.078 (3)	0.0413 (19)	0.043 (2)	0.0131 (19)	−0.0050 (18)	−0.0011 (15)
C18	0.046 (2)	0.067 (3)	0.071 (3)	0.0168 (19)	−0.0048 (18)	−0.002 (2)
N1	0.0448 (15)	0.0336 (13)	0.0361 (14)	−0.0177 (11)	−0.0047 (11)	0.0052 (11)
C17	0.0342 (18)	0.060 (2)	0.071 (3)	−0.0095 (16)	−0.0053 (17)	−0.0001 (19)
N3	0.0292 (12)	0.0327 (13)	0.0393 (14)	−0.0066 (10)	−0.0069 (10)	−0.0016 (10)
N4	0.0349 (14)	0.0403 (15)	0.0615 (18)	−0.0121 (12)	−0.0109 (12)	0.0156 (13)
O1	0.0258 (9)	0.0377 (11)	0.0256 (9)	−0.0075 (8)	0.0001 (7)	0.0014 (8)
O2	0.0420 (12)	0.0356 (11)	0.0309 (11)	−0.0135 (9)	−0.0033 (9)	0.0007 (9)
O3	0.0514 (13)	0.0489 (14)	0.0410 (13)	−0.0034 (11)	−0.0087 (10)	−0.0180 (11)
O4	0.0323 (12)	0.0397 (12)	0.0567 (14)	0.0075 (9)	−0.0049 (10)	−0.0215 (11)
Zn1	0.0272 (2)	0.0271 (2)	0.0254 (2)	0	−0.00199 (16)	0

### Geometric parameters (Å, °)

**Table:**

C1—O1	1.307 (3)	C13—H13	0.93
C1—C6	1.421 (4)	C14—O2	1.248 (3)
C1—C2	1.434 (4)	C14—N1	1.348 (3)
C2—C3	1.395 (4)	C15—N1	1.455 (4)
C2—C14	1.486 (4)	C15—C16	1.514 (4)
C3—C4	1.373 (4)	C15—H15A	0.97
C3—H3	0.93	C15—H15B	0.97
C4—C5	1.378 (4)	C16—N2	1.336 (4)
C4—H4	0.93	C16—C17	1.383 (4)
C5—C6	1.391 (4)	N2—C20	1.344 (4)
C5—H5	0.93	C20—C19	1.365 (5)
C6—C7	1.495 (4)	C20—H20	0.93
C7—O3	1.240 (3)	C19—C18	1.361 (6)
C7—N3	1.332 (4)	C19—H19	0.93
C8—N3	1.442 (4)	C18—C17	1.371 (5)
C8—C9	1.514 (4)	C18—H18	0.93
C8—H8A	0.97	N1—H1	0.86
C8—H8B	0.97	C17—H17	0.93
C9—N4	1.332 (4)	N3—H3A	0.86
C9—C10	1.377 (4)	O1—Zn1	1.9772 (18)
C10—C11	1.382 (4)	O2—Zn1	2.1572 (19)
C10—H10	0.93	O4—Zn1	2.149 (2)
C11—C12	1.368 (5)	O4—H24	0.85
C11—H11	0.93	O4—H23	0.87
C12—C13	1.374 (5)	Zn1—O1^i^	1.9772 (18)
C12—H12	0.93	Zn1—O4^i^	2.149 (2)
C13—N4	1.338 (4)	Zn1—O2^i^	2.1572 (19)
			
O1—C1—C6	120.1 (2)	C16—C15—H15A	109.3
O1—C1—C2	122.3 (2)	N1—C15—H15B	109.3
C6—C1—C2	117.5 (2)	C16—C15—H15B	109.3
C3—C2—C1	119.3 (2)	H15A—C15—H15B	108
C3—C2—C14	119.5 (2)	N2—C16—C17	121.2 (3)
C1—C2—C14	121.2 (2)	N2—C16—C15	117.5 (3)
C4—C3—C2	122.1 (3)	C17—C16—C15	121.3 (3)
C4—C3—H3	118.9	C16—N2—C20	117.6 (3)
C2—C3—H3	118.9	N2—C20—C19	123.7 (3)
C3—C4—C5	119.3 (3)	N2—C20—H20	118.2
C3—C4—H4	120.4	C19—C20—H20	118.2
C5—C4—H4	120.4	C18—C19—C20	118.6 (3)
C4—C5—C6	121.3 (3)	C18—C19—H19	120.7
C4—C5—H5	119.3	C20—C19—H19	120.7
C6—C5—H5	119.3	C19—C18—C17	118.8 (4)
C5—C6—C1	120.4 (2)	C19—C18—H18	120.6
C5—C6—C7	115.9 (2)	C17—C18—H18	120.6
C1—C6—C7	123.7 (2)	C14—N1—C15	124.6 (3)
O3—C7—N3	121.1 (3)	C14—N1—H1	117.7
O3—C7—C6	121.0 (3)	C15—N1—H1	117.7
N3—C7—C6	117.8 (2)	C18—C17—C16	120.1 (3)
N3—C8—C9	115.3 (2)	C18—C17—H17	119.9
N3—C8—H8A	108.4	C16—C17—H17	119.9
C9—C8—H8A	108.4	C7—N3—C8	122.0 (3)
N3—C8—H8B	108.4	C7—N3—H3A	119
C9—C8—H8B	108.4	C8—N3—H3A	119
H8A—C8—H8B	107.5	C9—N4—C13	117.6 (3)
N4—C9—C10	122.3 (3)	C1—O1—Zn1	130.85 (16)
N4—C9—C8	113.4 (2)	C14—O2—Zn1	127.87 (18)
C10—C9—C8	124.3 (3)	Zn1—O4—H24	121
C9—C10—C11	119.2 (3)	Zn1—O4—H23	128
C9—C10—H10	120.4	H24—O4—H23	96
C11—C10—H10	120.4	O1—Zn1—O1^i^	175.44 (11)
C12—C11—C10	118.9 (3)	O1—Zn1—O4^i^	85.97 (8)
C12—C11—H11	120.5	O1^i^—Zn1—O4^i^	97.42 (8)
C10—C11—H11	120.5	O1—Zn1—O4	97.42 (8)
C11—C12—C13	118.3 (3)	O1^i^—Zn1—O4	85.97 (8)
C11—C12—H12	120.8	O4^i^—Zn1—O4	84.55 (12)
C13—C12—H12	120.8	O1—Zn1—O2	84.29 (7)
N4—C13—C12	123.6 (3)	O1^i^—Zn1—O2	92.61 (8)
N4—C13—H13	118.2	O4^i^—Zn1—O2	168.82 (8)
C12—C13—H13	118.2	O4—Zn1—O2	91.26 (8)
O2—C14—N1	120.2 (3)	O1—Zn1—O2^i^	92.61 (8)
O2—C14—C2	124.2 (2)	O1^i^—Zn1—O2^i^	84.29 (7)
N1—C14—C2	115.7 (2)	O4^i^—Zn1—O2^i^	91.26 (8)
N1—C15—C16	111.5 (3)	O4—Zn1—O2^i^	168.82 (8)
N1—C15—H15A	109.3	O2—Zn1—O2^i^	94.67 (12)
			
O1—C1—C2—C3	179.9 (2)	C17—C16—N2—C20	0.0 (5)
C6—C1—C2—C3	−2.4 (4)	C15—C16—N2—C20	179.5 (3)
O1—C1—C2—C14	−1.7 (4)	C16—N2—C20—C19	−1.0 (5)
C6—C1—C2—C14	176.1 (2)	N2—C20—C19—C18	1.0 (6)
C1—C2—C3—C4	1.4 (4)	C20—C19—C18—C17	0.0 (6)
C14—C2—C3—C4	−177.1 (3)	O2—C14—N1—C15	2.9 (4)
C2—C3—C4—C5	0.5 (5)	C2—C14—N1—C15	−177.0 (3)
C3—C4—C5—C6	−1.3 (5)	C16—C15—N1—C14	−127.1 (3)
C4—C5—C6—C1	0.2 (4)	C19—C18—C17—C16	−1.0 (6)
C4—C5—C6—C7	−178.5 (3)	N2—C16—C17—C18	1.0 (5)
O1—C1—C6—C5	179.4 (2)	C15—C16—C17—C18	−178.5 (3)
C2—C1—C6—C5	1.6 (4)	O3—C7—N3—C8	6.7 (4)
O1—C1—C6—C7	−1.9 (4)	C6—C7—N3—C8	−174.4 (2)
C2—C1—C6—C7	−179.8 (2)	C9—C8—N3—C7	−83.3 (4)
C5—C6—C7—O3	−10.8 (4)	C10—C9—N4—C13	1.4 (5)
C1—C6—C7—O3	170.5 (3)	C8—C9—N4—C13	−178.6 (3)
C5—C6—C7—N3	170.3 (2)	C12—C13—N4—C9	−0.8 (6)
C1—C6—C7—N3	−8.4 (4)	C6—C1—O1—Zn1	151.87 (19)
N3—C8—C9—N4	−176.5 (3)	C2—C1—O1—Zn1	−30.4 (4)
N3—C8—C9—C10	3.5 (5)	N1—C14—O2—Zn1	168.55 (19)
N4—C9—C10—C11	−0.3 (5)	C2—C14—O2—Zn1	−11.5 (4)
C8—C9—C10—C11	179.7 (3)	C1—O1—Zn1—O4^i^	−143.2 (2)
C9—C10—C11—C12	−1.4 (5)	C1—O1—Zn1—O4	−59.2 (2)
C10—C11—C12—C13	1.9 (5)	C1—O1—Zn1—O2	31.3 (2)
C11—C12—C13—N4	−0.8 (6)	C1—O1—Zn1—O2^i^	125.8 (2)
C3—C2—C14—O2	−159.3 (3)	C14—O2—Zn1—O1	−9.8 (2)
C1—C2—C14—O2	22.2 (4)	C14—O2—Zn1—O1^i^	173.6 (2)
C3—C2—C14—N1	20.6 (4)	C14—O2—Zn1—O4^i^	19.8 (6)
C1—C2—C14—N1	−157.9 (2)	C14—O2—Zn1—O4	87.6 (2)
N1—C15—C16—N2	−127.8 (3)	C14—O2—Zn1—O2^i^	−101.9 (2)
N1—C15—C16—C17	51.7 (4)		

Symmetry codes: (i) −*x*, *y*, −*z*+1/2.

### Hydrogen-bond geometry (Å, °)

**Table:**

*D*—H···*A*	*D*—H	H···*A*	*D*···*A*	*D*—H···*A*
N3—H3A···O1	0.86	1.93	2.623 (3)	136
N1—H1···N4^ii^	0.86	2.20	3.007 (4)	155
O4—H24···O3^iii^	0.85	1.87	2.712 (3)	174
O4—H23···N2^iv^	0.87	2.03	2.879 (3)	163

Symmetry codes: (ii) *x*+1/2, *y*+1/2, *z*; (iii) −*x*, −*y*, −*z*; (iv) −*x*+1/2, *y*−1/2, −*z*+1/2.
