# Diaqua­{2,6-bis­[*N*-(2-pyridinylmeth­yl)­carbamo­yl]­phenolato-κ^2^ *O* ^1^,*O* ^2^}zinc(II). Corrigendum

**DOI:** 10.1107/S160053680803537X

**Published:** 2008-10-31

**Authors:** Chomchai Suksai, Sarayut Watchasit, Thawatchai Tuntulani, Chaveng Pakawatchai

**Affiliations:** a Department of Chemistry, Faculty of Science, Burapha University, Chonburi 20131, Thailand; b Department of Chemistry, Faculty of Science, Chulalongkorn University, Bangkok 10330, Thailand; c Department of Chemistry, Faculty of Science, Prince of Songkla University, Songkhla 90112, Thailand

## Abstract

The chemical name in the title of the paper by Suksai, Watchasit, Tuntulani & Pakawatchai [*Acta Cryst.* (2008), E**64**, m884–m885] is corrected.

## Abstract 2

Corrigendum to *Acta Cryst.* (2008), E**64**, m884–m885.

In the paper by Suksai, Watchasit, Tuntulani & Pakawatchai [*Acta Cryst.* (2008), E**64**, m884–m885], the chemical name in the title is incorrect. The correct title should be ‘Diaqua­bis{2,6-bis­[*N*-(2-pyridinylmeth­yl)­carbamo­yl]­phenolato-κ^2^ *O* ^1^,*O* ^2^}zinc(II)’.

